# Dr. Brewer’s Essay

**Published:** 1870-02

**Authors:** J. W. Brewer


DR. BREWER'S ESSAY.

MR. PRESIDENT.—As a member of this Association, I have your permission to offer for your consideration a few desultory remarks. It is, indeed a favor and privilege I duly appreciate; and, allow me to say, that I consider it a pleasant duty to be performed. Not that I expect to overwhelm or astound you with new or entirely original matter; on the contrary, my remarks may even seem dull and devoid of general interest to the practitioner; but, permit me, gentlemen, to indulge in the hope that such will not be the case. I take it for granted, that we are not only progressive, but kindly indulgent—indulgent to the faults and weakness of others—progressive in spirit and practice. If we fail to reach the summit of our ambition at a bound, we may, by close application and a steady perseverence in the right direction, win and merit the reward and approbation of our fellow-men. In accordance, Mr. President, to the expressed desire of the Chairman of the Committee on Therapeutics and *Materia Medica,* I am to confine my remarks, almost entirely, to that branch of our science. I think I may safely hazard the assertion, that it is a subject replete and full of interest to all parties concerned and interested in the welfare and advancement of medical science. Therapeutics, as we understand it, is the application, in general, of remedies in disease. I will refrain, however, from entering upon a learned dissertation. So far as relates to a medical definition of the terms, therapeutics and pharmacology, this, indeed, has been fully and ably accomplished by Professor Wood, in his excellent work upon therapeutics and *materia medica;* and, upon this point, I doubt not, you are fully posted. Of the utility and importance of a thorough knowledge and just appreciation of therapeutics and *materia medica*, to the practitioner, there cannot be a reasonable doubt. So fully impressed am I of this impartant fact, that I deem it essentially and eminently necessary that the well-educated physician be thoroughly posted and fully alive to the importance of a just and correct appreciation of the therapeutic applicability of remedies in disease. In fact, the absence of knowledge, on this point, would, doubtless, permit us to drift hopelessly in the turbid waters of empiricism, and to be lost amid the rocks and breakers of quackish pretension.

I desire, however, to state, before entering fully upon the points to which I solicit your attention, that I do not propose to discuss at this meeting the merits or individuality of any particular drug. The limited space allowed me precludes the possibility of entering into a detailed account of the various properties and medicinal value of the countless legion of remidies contained in the pharmacopœia. My remarks, at this time, will be of rather a general character, embracing many points of interest, and, as it occurs to me, suggestions of practical importance to us all.

It is doubtless understood by many present, that I am not actively engaged in the practice of medicine, and that for the last 20 years, I have devoted the principal part of my time to the dispensing of drugs. It is, therefore, but a reasonable presumption that I have a moderate experience in this matter.

Having, then, experience, both as a physician and druggist, it is not strange, then, that I should discover the necessity and vital importance of harmony, good-will, and perfect undertanding existing between the dispenser and physician. Indeed, the connection between them should be a close and intelligent intimacy. It is, without doubt, preëminently and essentially incumbent upon the practitioner, that in his choice and selection of the pharmaceutist, who is to compound his remedies, that he use the utmost precaution and judgment. Now, this advice may seem, at first sight, as superfluous; but, upon giving the subject a moment's serious reflection, you cannot, I am confident, gainsay this practical fact. The compounder of your prescriptions should be a man of sound judgment, large experience, and a thorough and accomplished pharmaceutist—possessing, honesty, intelligence, and integrity in an eminent degree—a worthy member and graduate of some acknowledged school of pharmacy, and who has an intelligent and just appreciation of a strict adherence and observance of pharmaceutical requirements. Now, the absence or lack of these all-important requirements will, most surely, sooner or later, lead to disappointment, annoyance, and perplexity, not only to the prescriber, but to the patient and all concerned.

Any deviation, adulteration, substitution, or ignorant rendition of a prescription, however slight, will, most surely defeat the main object sought to be accomplished by the practitioner. That there are many departures from a strict observance and an honest adherence to the pharmacopœia, we all must concede to be a lamentable fact—the pharmaceutists themselves acknowledge this to be so—and for which there cannot, or ought not to be, any excuse. Let it be understood, however, that I do not propose to make a wholesale onslaught upon the character and professional standing of our pharmaceutists, or to cast a doubt upon the honesty and purity of their intentions; on the contrary, I take pleasure in asserting that there are many, very many, honorable exceptions to this loose and dishonest following of the pharmacopœia. As a class, I think, I may safely aver that the druggists of Illinois will compare favorably with the druggists in any other portion of the United States; not only as to ability and capacity, but also for an honest and honorable commercial integrity, and who are animated with an ardent desire to further the interests and promote a radical reform in the dispensing of pure and reliable drugs. A desire for improvement and progression in this matter is manifestly evident, from the exertions that are being daily put forth, by many of our leading pharmaceutists, which, if met and sustained by corresponding and reciprocal exertions on the part of the practitioner, cannot fail in satisfactory results. In short, without a full and hearty concurrence and evident cooperation of the physicians, and the sustaining influence of the community, the exertions being put forth in this movement must ever fall short of the mark.

It is ever an arduous undertaking for honesty to keep pace with roguery, either in invention or application. Genius is always on the alert for original developments, among the shrewd and gifted pretenders, and is ever engaged in a pertinacious search for new resources.

On the contrary, integrity sits down content with its present means, and is only spurred to novelty when set at defiance by the mortifying evidences of the advance of others in the race of progress.

It is, therefore, self-evident that me must ever be on the alert, lest, through supineness and want of activity, we lose the race.

I am constrained to acknowledge the fact, that in many instances, community, and even physicians themselves, are too apt to encourage the traffic in cheap drugs, thereby tacitly, if not directly, sanctioning the adulteration of drugs.

I hope, for the credit of the profession, that the doctors present will kindly pardon and indulge me, while I offer a few practical remarks in regard to this matter, and for the veracity of which almost every druggist in the land will sustain me.

It is the constant and increasing desire, almost amounting to a mania, on the part of the patient or his friends, to get their prescriptions compounded for nothing. The constant and persistent annoyance the pharmaceutists meet with in this respect is, in fact, most discouraging, and unless met with prompt and sterling integrity on the part of the dispenser, must necessarily, in its results, produce want of confidence in the prescriber, as well as a lack of faith in the potency of remedies.

This practice is susceptible of another very serious and damaging result. It opens wide the door to irresponsible, ignorant, and dishonest competition, and makes easy the entrance of persons who are ever ready and willing to traffic upon the afflictions of others. It gives employment and competency to the unscrupulous and incompetent pretender, whose active genius is constant and ever on the alert, in the art of adulteration, dilution, and substitution.

Have physicians, I would inquire, given this matter the careful and scrupulous investigation it deserves? do they recognize the urgent necessity of prompt, stern, and absolute reform, and a hearty coöperation with that portion of druggists who are ready and willing to unite with them, in this much-needed reformation? To do full justice to the profession, I freely confess—permit me to say, that I do so, with much pride and satisfaction—that I have ever found the great majority of intelligent physicians on the side of progression, and fully alive to the great importance of this matter. I have within the last few years conversed freely with many practitioners, upon this subject, and they, with one accord, assure me that they are ever ready to aid and assist in elevating the professional standard of the legitimate pharmaceutist. It is also with pride that I notice the deep and abiding interest that many of our eminent pharmaceutists are taking in this all-important subject. I could call your attention to many able and well-written articles on this matter, written by earnest and worthy men; but it were needless. Suffice it to say, that this subject is attracting the notice of many of our leading physicians and chemists, and every effort is being put forth to the furtherance of this purpose. Societies and associations are being formed for the protection and elevation of the legitimate pharmaceutist, and a determined effort is manifested to purge and drive out all unreliable and irresponsible pretenders. It is even suggested that severe and stringent legislative enactments be enforced.

How far, however, these extreme measures would be sustained by the public remain to be seen. I have, however, occupied your considerate attention in this matter, perhaps, too long, although, I hope not. I consider my time well spent, if I but succeed in arresting your attention for a time to this subject; may I indulge the hope, that henceforward the members of this Association will be ever ready to extend the hand of fellowship and to aid and sustain, by their influence, the professional character of the worthy and legitimate pharmaceutist.

As I have said before, I will not discuss the merits of any particular remedy, at this meeting; it being as highly important, in this age of progression, that our attention be directed to matters of general interest, having a direct bearing, and exercising a controlling influence, upon medical science and experience.

The numerous abuses, false theories, and absurd dogmas existing, and which are in daily conflict, and intimately connected, with therapeutics and pharmacology, needs, at least, a passing notice. I desire, now, to say a few words, in regard to the multitudinous host of new remedies and preparations foisted upon the attention of the profession, by the Nichols, Tildens, Thayers, Duffields, Sargents, Merrills, Garrisons, and a host of others.

These gentlemen have been indefatigable in their efforts and have exhibited ingenuity ard activity to an astonishing degree, in the magnitude of their preparations. In fact, so numerous and prolific are the various compounds offered for our inspection, that even to test them, we will be obliged to almost entirely abandon the good old time-honored theories of our fathers. Will this pay in the end? Ah! here, gentlemen, you have an ample field for reflection and also for the exercise of your patient and profound judgment. The astonishing and persistent versatility with which these learned gentlemen point out the wonderful remedial and curative properties of their compounds, is almost beyond conception, and gives ample opportunity for the exercise of our credulity.

While I deprecate their almost patent medicine style of oratory, in the recommendation of their various compounds, and while, also, I doubt the efficacy of many of their preparations, yet, in all candor, and to do full justice to these gentlemen, I am constrained to admit, that in their efforts, they have produced many meritorious and worthy combinations, eminently entitled to our confidence; and the only difficulty in the way, as it seems to me, is the ability to distinguish between the good and the worthless. And this, gentlemen, is no light task, when you take into consideration the endless list of alkaloids, resinoids, fluid extracts, concentrated preparations, syrups, elixirs, etc., etc.

In my experience as physician and druggist, I have been induced, through the energetic recommendations of the manufacturers, to give many of these preparations a trial. I have done so honestly and with a hope that they would meet the indication. The fluid extracts, for instance, I may safely say, I have tested most thoroughly, inasmuch as I was particularly anxious, as a druggist, that they should prove themselves to be all that was so confidently claimed for them; it being a great saving in time and labor in making syrups, tinctures, and infusions from them. After a careful and patient trial, in my experience, I cannot say that the results are entirely satisfactory. Indeed, I have, in a great measure, abandoned this method of preparing my tinctures and syrups, and have resorted once more, at the risk of being classed by these scientific gentlemen as an old fogy, to the old plan, as directed by the United States Dispensatory.

Now, as to the possibility of making and keeping up the standard of the fluid extracts to the required point, there cannot be a reasonable doubt. There are, I believe, one or two manufacturers who keep these preparations up to the standard, but do not, I am sorry to say, meet with the support due them. The fact that a preponderance of the fluid extracts now thrust upon the market are mere dilutions, scarcely representing the strength of common tinctures, has been clearly proven to be the case in New York, as well as in other places.

Here we recognize our old and steadfast enemy *adulteration*, who is ever ready and always active in his exertions to pander to the diseased appetite of the public for cheap drugs. Our only remedy, then, is a steadfast and strict adherence to the pharmacopœia. How is this to be effected? I think it can be accomplished by giving attention to the following facts:—

In May, 1870, the next decennial convention of delegates from the incorporated medical and pharmaceutical bodies of the United States meets in Washington, for the purpose of taking the usual measures to effect the fifth revision of the pharmacopœia. It is of great importance to both physicians and pharmaceutists that this revision should be wisely considered and thoroughly executed, so that the pharmacopœia may command the respect and recognition in practice, so necessary to its usefulness. In the absence of any legal power to compel the adoption of its formula, it is worthy of the earnest consideration of the medical profession and of all well-informed pharmaceutists, to devise some plan by which a more general recognition of its authority may be effected.

Now, the United States Dispensatory is to be found in every shop, yet, nevertheless, there is a disposition on the part of many to depart from officinal rules, when to obey them is more troublesome or more expensive than some short cut process, that they may have devised. This is especially apt to be the case in what are termed the galenical preparations, as extracts, fluid extracts, elixirs, syrups, etc., etc.; the deviation often so alters the appearance and composition of the results, as to cause doubt and distrust, on the part of well-informed physicians.

The first step toward a reform, in this matter, is to make the pharmacopœia, of 1870, as nearly perfect as possible. Let its formulas, especially those most certain to be used by the dispensing pharmaceutist, be marked for its simplicity and directness of their manipulations and for the excellence and attractive appearance of their results, when these qualities are not incompatible; always giving the preference to intrinsic rather than apparent value.

The second step must be taken by physicians, in their society capacity, chiefly—the American Medical Association is the medical body best able to exert a universal influence within our national boundaries. In promoting the practical recognition of the United States Pharmacopœia. That Association meets the same week, in the same city, with the Pharmacopœal Convention. And, now, united, North and South will be in the best condition to exert a lasting influence on the future standing of the pharmacopœia, by lending its entire influence in favor of officinal preparations, made by officinal formula. This may be done, first, by declaring its convictions in a few well-drawn resolutions, directed to its members; and secondly, by urging forcibly on the attention of each of the subordinate State and County Medical Associations, to examine the edition, when published, through a competent committee, recommending their members to become acquainted with its officinal names and new preparations, and peremptorily refuse to prescribe A, B, and C's compounds, which profess to be better, until the officinal have been found wanting. It is often said that medical men are easily persuaded into using medical novelties; that advertising is the true secret of getting up the reputation of medicines among physicians.

This idea is so well recognized by manufacturers, that many instances occur of their publishing journals, at merely nominal prices, in order to introduce their products. It, therefore, appears to be true, however humilitating it may be to the medical profession, that many of its members are "led by the nose," as it were, through the agency of quick-witted advertisers, on the covers of medical journals, and in circulars and pamphlets. The remedy for this evil is better professional care, better acquaintance with the *materia medica*, better knowledge of the preparations of the pharmacopœia, by actual inspection, so as to be able to tell, as far as sensible properties permit, what their patients are getting.

We do not believe the possession and exercise of this knowledge, on the part of the physicians, will unfavorably influence legitimate pharmacy, but will increase the demand for regular preparations, at the expense of the many fancy novelties now inundating the shops.

It may seem that I am needlessly severe. It may be so; but it cannot be said that I have not given my vote to merit, uninfluenced by malice, prejudice, or from any selfish motives.

I am willing to concede to these energetic chemists and manufacturers all they deserve. I do not desire to be misconstrued in my remarks concerning their compounds.

They have taught us many valuable lessons: the elegance of many of their preparations is worthy of emulation, and have proven themselves to be, in many cases, a great blessing to the suffering.

If it were possible that they maintain strict integrity, as to strength and purity, and a steady adherence to their published formula, then, indeed, our confidence would not be misplaced and our faith more abiding. The great evil to be deplored is not the absence of knowledge or skill, or want of elegance of their compounds, but in the fact that many of the manufacturers lack that high and sterling integrity, so vital in the maintenance of purity and reliability in their preparations; and, I regret to say, that they too readily yield to the baneful influences by which they are surrounded—influences to which, in their lust for competency, they are too apt to succumb. Their great desire is to undersell their neighbors, to introduce something cheaper—this is the great source of the evil. The almost incessant clamor for cheap drugs, by everybody, is productive of, at least, one result, and that is, the desire to supply the want; and, you may rest assured, it is too often done. Does an agent from some new manufacturer make his advent into your place of business? his very first greeting is: "I have here a very nice article, I wish to present for your inspection: our facilities for manufacturing are such, that we can offer you this article at a much less cost or price than it has ever been offered before." Now, what are his facilities? I will tell you—they are, beyond all doubt, facilities for dilution—can you doubt this? Now, for instance, the article of ipecac, perfectly pure, has a market value; does any one offer it for less, you may safely conclude they are not doing a legitimate business. I might add numerous instances to substantiate this position, but it is not necessary. If we had time and space, we might also discuss the feasibility of many of the proposed laws now being introduced, and under discussion, by the pharmacists themselves, at their regular meetings—laws that ere long will, doubtless, be presented to our legislative bodies for enactment.

Now, gentlemen, whether we are willing or not to lend our aid to this much-needed reformation, nevertheless, the ball is in motion, and good will come of it.

I do not claim to be the pioneer in this movement; wiser heads than mine have long seen the urgent need for swift and absolute reform. I have been in the drug business too long not to know, that the time and attention bestowed upon this matter by the medical profession will reward them richly.

They have the power, let them exercise it; encourage none but competent men, and, even here, give them to understand that you hold them to strict accountability for a correct rendition and interpretation of your prescriptions, and that you will in no event submit to any substitution or dilution, that may be made, in order to satisfy the demand of some parsimonious patient for a cheap compound.

Gentlemen, I might continue this subject to an indefinite length, but it were needless; you have all and every one, doubtless, given this matter ample consideration, long before this, at least, I would fain believe that such is the case.

And, now, gentlemen, in conclusion, I hope my remarks will meet with your hearty approval.

Let physicians act as a unit in this important matter, now that the pharmaceutists are fully alive to the necessity of improvement in therapeutical integrity.

Let your influence be such as to encourage and sustain them in their laudable undertaking. Avoid criminations and recriminations. Aid the worthy and condemn the unworthy. Let merit be sustained however humble the condition. By pursuing this course you will soon be satisfied as to the results.

Permit me to tender you my thanks for the kind, patient, and indulgent hearing you have given me, and to indulge the hope that I have not trespassed upon your valuable time, and, also, that we are upon the eve of a bright future. A future pregnant with research, industry, and patient investigation—a future of confidence, trust, and a just appreciation of the friendly and intimate relation that should exist between the physician and pharmaceutist.

J. W. BREWER, M.D.

